# Diagnostics for Typhoid Fever: Current Perspectives and Future Outlooks for Product Development and Access

**DOI:** 10.1093/ofid/ofad120

**Published:** 2023-06-02

**Authors:** Jyotshna Sapkota, Tamalee Roberts, Buddha Basnyat, Stephen Baker, Lee M Hampton, Sabine Dittrich

**Affiliations:** FIND, the Global Alliance for Diagnostics, Geneva, Switzerland; Department of Microbiology, Nepal Medical College, Kathmandu, Nepal; Lao-Oxford-Mahosot Hospital-Wellcome Trust Research Unit, Microbiology Laboratory, Mahosot Hospital, Vientiane, Lao People’s Democratic Republic; Centre for Tropical Medicine and Global Health, Nuffield Department of Medicine, University of Oxford, Oxford, United Kingdom; Oxford University Clinical Research Unit, Kathmandu, Nepal; University of Cambridge School of Clinical Medicine, Cambridge Biomedical Campus, Cambridge, United Kingdom; Department of Medicine, University of Cambridge School of Clinical Medicine, Cambridge Biomedical Campus, Cambridge, United Kingdom; Gavi, the Vaccine Alliance, Geneva, Switzerland; FIND, the Global Alliance for Diagnostics, Geneva, Switzerland; Centre for Tropical Medicine and Global Health, Nuffield Department of Medicine, University of Oxford, Oxford, United Kingdom; Deggendorf Institute of Technology, European Campus Rottal-Inn, Pfarrkirchen, Germany

**Keywords:** point of care tests, rapid diagnostic tests, *Salmonella* Typhi, typhoid diagnostics

## Abstract

Typhoid is an enteric disease caused by *Salmonella* Typhi. Like many febrile illnesses, typhoid presents with nonspecific symptoms. In routine healthcare settings in low- and middle-income countries, typhoid fever is suspected and treated empirically. Though many diagnostic tests are available for typhoid diagnosis, there are currently no diagnostic tests that meet ideal requirements for sensitivity, specificity, speed, and cost-effectiveness. With introduction of typhoid conjugate vaccine, it is essential to explore the current and future typhoid approach in the context of use case and access to ensure their utilization for disease control.

Regional estimates of the burden of typhoid fever cannot be measured accurately without improved disease diagnostics; this lack of diagnostics and data impacts the ability of governments to plan and appropriately intervene. Given the need for disease control, funding typhoid diagnostic capacity, including availability and use of improved typhoid test kits, should be increased, especially where the incidence of typhoid is unknown [1]. Challenges regarding typhoid diagnostics may also impact the implementation of new-generation typhoid vaccines in endemic regions due to lack of surveillance tools [2]. Multidrug resistance (MDR) has been spread intercontinentally due to an increase in travel connectivity, affecting those living in endemic regions and travelers alike [3, 4]. Notably, multidrug-resistant and fluoroquinolone-resistant variants of *Salmonella enterica* subspecies *enterica* serovar Typhi (*S.* Typhi) may be associated with more severe disease with potentially adverse outcomes, therefore creating clinical management challenges [3]. The spread of drug-resistant organisms as well as an expected reemergence of typhoid in currently nonendemic settings due to climate change make improved diagnostics key for tracking incidence to inform public health policy in additional to ensuring individual patients get appropriate treatment. Here, we aim to review gaps in the diagnostic landscape for typhoid and explore new technological and access developments that could improve the diagnostic landscape in the future and in the context of existing target product profile drafts. We explore different areas of typhoid diagnostic challenges, including current shortcomings in an example setting, expanding understanding of existing rapid diagnostic tests (RDTs), the future of diagnostics as part of surveillance, and access-related considerations that aim to improve the availability of quality diagnostic tests in the future.

## CHALLENGES IN TYPHOID DIAGNOSIS: EXAMPLE FROM LAOS

The Lao People’s Democratic Republic (Laos) is a landlocked country in Southeast Asia with a population of approximately 7 million people [5]. Typhoid fever is endemic in Laos, but there are limited epidemiological data. In a hospital-based study examining blood cultures at Mahosot hospital in the capital, Vientiane, between 2000 and 2018, there were a total of 913 culture-confirmed typhoid cases from just over 60 000 blood cultures (∼1.5% positivity). Most cases originated in rural areas with the majority of patients recruited into research studies; there were limited specimen requests outside of these studies, particularly outside of the capital city [6].

These data suggest that the amount of typhoid in Laos is underestimated; detection relies on blood culture, with the laboratory capacity to process blood cultures being limited outside of Vientiane (capacity is increasing as a consequence of a UK Fleming Fund grant). Provincial hospital laboratories in the network perform manual blood cultures in-house and then send positive cultures, including suspected *S.* Typhi, to a central government or reference laboratory in Vientiane for identification or confirmation. The shipping process can take several days mostly due to infrastructure challenges (eg, lack of transportation/staff, unpassable roads) with blood culture bottles sitting at room temperature without any additional temperature regulation. This leads to isolates not being recovered in the reference laboratory in Vientiane due to high temperatures and transportation conditions. A previous study recorded temperatures as high as 41°C in a transportation box used for sample shipment [7]. The cost and availability of confirmation tests also impacts the diagnostic result, as high costs of identification reagents, antisera, and shipment may be prohibitive for laboratories in low- and middle- income countries (LMICs); for example, API 20E identification strips cost approximately US$6 per test and antisera cost approximately $3 per test and can take months to be shipped to the country. Blood culture is recognized to be the gold standard for typhoid diagnosis; however, it is a complex and expensive process as highlighted by the example from Laos. In this example use case, financial and logistic support can be provided due to research affiliation of the laboratories and even here things are not working in a way that provides reliable, high-quality data. The result of such imperfect data is the underestimation of typhoid cases nationally but also in the areas with access to research network.

This example from Laos shows that without local capacity and appropriate (cheap, long shelf life, simple to use) diagnostic tests, provincial hospitals have to continue to rely on sample transport over large distances. This type of centralized testing leads to lost time and quality and as a result clinicians are inclined to skip the laboratory sample and rather go ahead with treatments without laboratory confirmation. However, realistically, blood or bone marrow cultures, which are highly specific and considered the gold standard, are not suitable for use outside of well-established centers and not truly close to the point of care. This lack of accurate diagnostic testing has a negative impact on patient care and reliable incidence data.

## THE LACK OF SUITABLE RAPID DIAGNOSTIC TESTS

Ultimately to most of the challenges described in the case study, well-performing, high-quality tests are needed to be performed in a decentralized manner at the point of contact and not at a central facility that requires sample shipment. While many tests exist and are used at point of care (POC) by minimally trained staffs, unfortunately few meet the “well performing” or “high quality” bar that is equally essential. Various RDTs and different forms of the Widal test are commonly used in health facilities around the world to diagnose typhoid. These tests are cheap and simple, do not require sophisticated laboratories, and deliver results in a shorter time frame than blood culture, making them very popular. However, such tests lack sensitivity and specificity and thus are not of sufficient accuracy to replace blood culture as the main diagnostic approach for typhoid fever. The Widal test is the most used test to diagnose typhoid despite a low performance (sensitivity range, 57%–74%; specificity range, 43%–83% [8]) reported in several studies. A 2017 Cochrane review summarized the evidence on diagnostic accuracy of available RDTs for enteric (typhoid) fever (mostly TUBEX, Typhidot, and KIT Test-It) [9]. The result of the meta-analysis found TUBEX to have an average sensitivity of 78% and a specificity of 87%; Typhidot had an average sensitivity of 84% and a specificity of 79%, and Test-It (KIT) was found to have an average sensitivity of 69% and a specificity of 90% [8]. Numerous studies had been conducted to assess commercial diagnostic tests for typhoid [8]; however, key opinion leaders highlighted that these studies are difficult to compare due to a lack of comparable case definitions and a lack of geographical diversity. To address these data shortcomings, FIND established a head-to-head comparison of commercial typhoid tests and simultaneously generated a sample set that could be used in the evaluation of emerging technologies [10]. Typhoid positives and negatives were analyzed in both regions with 205 positives and 205 negatives from Asia and 59 positives and 59 negatives from Africa. Nine different RDTs were evaluated against blood culture as the reference standard. The tests used were SD Bioline *Salmonella* Typhi immunoglobulin G (IgG)/immunoglobulin M (IgM), Typhidot Rapid IgG/IgM, Enterocheck WB, Test-It Typhoid IgM, CTK Typhoid IgG/IgM Combo Rapid Test CE, Spectrum Typhoid IgG/IgM Rapid cassette, TUBEX-TF, Diaquick S.Typhi/Paratyphi antigen cassette, and the Widal test. The sensitivity values varied widely between the different tests, from 0% with the Diaquick antigen cassette to 78.8% with the IgG component of the CTK Typhoid IgG/IgM Combo Rapid Test CE. Overall, the study confirmed that no test currently meets the desired accuracy criteria [11] and diagnostic innovation is critical.

## THE FUTURE OF TYPHOID DIAGNOSTICS

While a variety of techniques are currently in use for the diagnosis of typhoid, no single technique satisfies the requirement for sensitivity and specificity while being rapid and cost-effective. This was again confirmed in the most recent data generated by FIND and partners [9], and the need for innovations was once again made obvious. However, future innovation for typhoid diagnosis should not only focus on disease diagnosis for immediate treatment purposes but also disease surveillance and the detection of carriers, to support public health interventions. Ultimately both aspects are different sides of the same coin and need to be advanced simultaneously to accelerate disease elimination as a whole.

RDTs using selected antigens such as the protein HlyE and sugars in the lipopolysaccharide are under investigation and exhibit some potential [12, 13]. Furthermore, studies using metabolomic platforms have sought to identify biomarkers specific to typhoid. Identifying a single or a combination of metabolites during the course of typhoid illness could provide several promising biomarkers [14–16]. Polymerase chain reaction (PCR)–based detection of typhoid in the blood generally shows poor sensitivity. Conventional to real-time PCR and nested and multiplex PCR using different targets have been used to diagnose typhoid with sensitivity ranges of 40%–100% [17]. However a more recent study using machine-learning algorithms to identify expression signatures of host-associated genes showed some promise. This study identified the transcripts of 5 key genes (*STAT1*, *SLAMF8*, *PSME2*, *WARS*, and *ALDH1A1*) that can differentiate enteric fever from other febrile illness; this approach may have some traction for a multipathogen diagnostic approach [18]. The latter 2 approaches might provide better value and may aid in identifying the cause of undifferentiated febrile disease (including typhoid) in resource-limited LMICs for better patient management. At this point, however, this is not yet the case as none of the existing tools meet the needs of resource-poor settings, both in terms of cost and performance [17]. A tool would have to be cheap and simple to use (akin to the GeneXpert) to really make it suitable for hospitals in lower-resource settings. While simple molecular tools to be used at the POC level were scarce pre-2020, after the scientific advancements and investments made linked to the severe acute respiratory syndrome coronavirus 2 (SARS-CoV-2) pandemic it might be more feasible to think about a workable POC device that can be used to identify a magnitude of possible fever causes [18]. Given the complexities of typhoid diagnosis in patients or carriers using simply accessible samples, public health agencies might have to resort to identifying the pathogens in the environment as a proxy for patients.

Molecular approaches look promising to detect *S.* Typhi in environmental samples. These methods are not meant as tools for healthcare workers to inform patient management so will not advance this area, yet they might open up a more promising area for public health surveillance, similar to cholera.

When thinking about and envisioning the next generation of diagnostic tools, it is critical that we do not confuse the different use cases and that we make sure the future Target Product Profiles account for all. As of now, the ideal approach unfortunately remains elusive as it needs to be low-cost and simple to use even when deployed for environmental surveillance. Looking toward a future with increased focus on typhoid, the most likely scenario is a combined approach where more high-tech approaches are developed by research and public health authorities and individual patient management remains to be guided by culture as well as improved RDTs.

## IMPROVED DIAGNOSTICS ACCESS TO IMPROVE VACCINE DELIVERY

Arguably, one of the biggest consequences of the current limitations in typhoid diagnostics and the resulting data gap on true prevalence is the ability of governments to determine whether and where to use typhoid conjugate vaccine (TCV). Two TCVs have been prequalified by the World Health Organization (WHO) [19]. The WHO Strategic Advisory Group of Experts recommends prioritizing TCV introduction in areas with high typhoid fever burden and areas with high prevalence of antimicrobial resistance (AMR) [20]. Although the introduction is good news for many countries, due to the lack of quality diagnostics both at the central (eg, blood culture or environmental surveillance data) or decentralized level (eg, reliable RDT data, as part of the surveillance data set), the same countries often struggle to justify the use of TCVs due to missing data. The lack of data links both to typhoid as well as AMR data, the latter also requiring microbiology facilities.

Since 2017, a TCV vaccine has been approved for funding support by Gavi, the Vaccine Alliance, and will be made available to eligible countries [21]. Liberia, Malawi, Nepal, Pakistan, and Zimbabwe have been approved for funding support and related TCV introduction support. Sixteen countries theoretically eligible for Gavi support do not have reliable typhoid surveillance data in the public domain (ie, blood culture confirmation, since at least 1995) [22]. In light of the outlined limitations of all currently available typhoid RDTs, the WHO recommends that blood culture be used as the preferred diagnostic test for guiding immunization program decisions [23]. Gavi is working to help make improved typhoid diagnostic tests available to countries eligible for Gavi funding support. Such improved tests should facilitate country efforts to scale up reliable typhoid diagnostic testing as part of multidisease surveillance systems, which in turn should lead to improved availability and use of information to ensure that immunization programs are more effective, efficient, and equitable [24].

## CONCLUSIONS

Reviewing the past, present, and future of typhoid diagnostic tests highlights that we really have not progressed rapidly since the introduction of the Widal test. There are many advances still to be made to enable the timely and reliable diagnosis of typhoid infections. Our overview of use cases ranging from patient management to environmental surveillance and vaccine allocation highlight the critical need for research and product development work. We argue that we are in the right period to solve these problems, with the ongoing SARS-CoV-2 pandemic showing the importance of diagnostics for disease mitigation. Additionally, the fact that Gavi is a committed ally for diagnostics is encouraging and may help to steer additional resources toward the development of pragmatic tools.

## Notes

***Financial support***. The study mentioned on “The lack of suitable rapid diagnostic tests” was conducted by FIND through DFID grant.

***Supplement sponsorship.*** This article appears as part of the supplement “Charting the Course to Meet the Challenges Ahead: Research and Developments on Typhoid and Other Invasive Salmonelloses” sponsored by the Coalition against Typhoid Secretariat, housed at the Sabin Vaccine Institute in Washington, DC and made possible by a grant from the Bill & Melinda Gates Foundation.
